# Association between Albumin-Bilirubin Grade and Non-Protein Respiratory Quotient in Patients with Chronic Liver Diseases

**DOI:** 10.3390/jcm8091485

**Published:** 2019-09-18

**Authors:** Ryo Takata, Hiroki Nishikawa, Hirayuki Enomoto, Kazunori Yoh, Yoshinori Iwata, Yoshiyuki Sakai, Kyohei Kishino, Naoto Ikeda, Tomoyuki Takashima, Nobuhiro Aizawa, Kunihiro Hasegawa, Noriko Ishii, Yukihisa Yuri, Takashi Nishimura, Hiroko Iijima, Shuhei Nishiguchi

**Affiliations:** Division of Hepatobiliary and Pancreatic disease, Department of Internal Medicine, Hyogo College of Medicine, Nishinomiya, Hyogo 663-8501, Japan; chano_chano_rt@yahoo.co.jp (R.T.); enomoto@hyo-med.ac.jp (H.E.); mm2wintwin@ybb.ne.jp (K.Y.); yo-iwata@hyo-med.ac.jp (Y.I.); sakai429@hyo-med.ac.jp (Y.S.); hcm.kyohei@gmail.com (K.K.); nikeneko@hyo-med.ac.jp (N.I.); tomo0204@yahoo.co.jp (T.T.); nobu23hiro@yahoo.co.jp (N.A.); hiro.red1230@gmail.com (K.H.); ishinori1985@yahoo.co.jp (N.I.); gyma27ijo04td@gmail.com (Y.Y.); tk-nishimura@hyo-med.ac.jp (T.N.); hiroko-i@hyo-med.ac.jp (H.I.); nishiguc@hyo-med.ac.jp (S.N.)

**Keywords:** chronic liver disease, npRQ, ALBI, liver cirrhosis, correlation

## Abstract

We sought to elucidate the relationship between albumin-bilirubin (ALBI) grade and non-protein respiratory quotient (npRQ) calculated by indirect calorimetry in chronic liver disease (CLD) patients (*n* = 601, median age = 63 years). Factors linked to npRQ < 0.85, which is reported to be an optimal cutoff point for the prognosis in liver cirrhosis (LC) patients, were also investigated using univariate and multivariate analyses. The median npRQ for all cases was 0.86. In total, 253 patients (42.1%) had npRQ < 0.85. The proportions of patients with npRQ < 0.85 in LC and non-LC patients were 51.9% (166/320) in LC patients and 31.0% (87/281) in non-LC patients (*p* < 0.0001). The median npRQ in ALBI grades 1, 2, and 3 for all cases were: 0.89, 0.85, and 0.82 (overall *p* < 0.0001). The proportions of patients with npRQ < 0.85 were 31.0% (71/229) in ALBI grade 1, 46.34% (152/328) in ALBI grade 2, and 68.18% (30/44) in ALBI grade 3 (overall *p* < 0.0001). In multivariate analyses of factors linked to npRQ < 0.85, ALBI grade 3 (*p* = 0.0095, hazard ratio = 3.242, ALBI grade 1 as a reference) was an independent predictor along with prothrombin time (*p* = 0.0139). In conclusion, ALBI grade can be a useful marker for npRQ in patients with CLDs.

## 1. Introduction

The liver plays a pivotal role in the nutrition metabolism and the storage of nutrients; it is deeply involved in the maintenance of serum blood glucose, gluconeogenesis from glycogen, amino acids and lactic acid, and the production of ketone bodies from fatty acids [1,2]. Liver cirrhosis (LC), which occurs after a long period of time due to persistent inflammation in the liver, is often complicated with protein-energy malnutrition (PEM) [1,2,3,4,5]. Protein malnutrition can be evaluated by serum albumin level, and energy malnutrition can be evaluated by testing the non-protein respiratory quotient (npRQ) using indirect calorimetry [6]. PEM is one of the representative complications seen in LC patients, and it can be linked to high morbidity and mortality in LC patients [1,2,3,4,5,6,7]. A previous report showed that protein malnutrition as defined by serum albumin < 3.5 g/dL and energy malnutrition as defined by npRQ < 0.85 were found in 61% and 43%, respectively, and PEM was found in 27% of LC patients (*n* = 294) [8]. RQs reflect which macronutrients are being metabolized; RQ level that approaches 0.7 indicates that lipids are being consumed, and RQ level that approaches 1.00 indicates that carbohydrates are largely being burned [9]. RQ level may exceed 1.00 during intensive exercise [9]. Previous studies reported that npRQ had significant correlation with percentage of arm circumference, percentage of arm muscle circumference, and free fatty acid (FFA) [10,11].

The albumin-bilirubin (ALBI) grade is a simple evaluation method for hepatic function, which is calculated by only serum albumin and total bilirubin, to overcome limitations of Child-Pugh classification in clinical settings [12]. That is, it includes several subjective items (ascites and encephalopathy) and interrelated items (ascites and serum albumin) [12]. After the introduction of the ALBI grading system, the good predictability of the ALBI grading system was confirmed in LC patients, irrespective of liver disease etiologies or the presence of hepatocellular carcinoma (HCC) in numerous clinical studies [13,14,15,16,17,18,19,20,21,22]. However, to our knowledge, the relationship between ALBI grade and npRQ in chronic liver disease (CLD) patients is largely unknown. This issue appears to be of clinical importance because indirect calorimetry is time-consuming and has high cost [23,24]. In the present study, we sought to elucidate this important research problem. Here, we present the current status of energy metabolism in Japanese CLD patients using a large sample.

## 2. Patients and Methods

### 2.1. Patients

A total of 601 CLD patients with data for npRQ available were admitted to our hospital between October 2005 and August 2018, and they were analyzed in this study. All analyzed patients had liver histological data (F0–F4), and 594 patients (98.8%) had bioimpedance analysis (BIA) data (skeletal muscle index (SMI) or extracellular water (ECW) to total body water (TBW) ratio). ALBI score was calculated and classified into three grades (ALBI grades 1, 2, and 3) as reported previously [12].

### 2.2. Measurement of npRQ by Indirect Calorimetry

Carbohydrates, lipids, and proteins taken into the body are metabolized to produce energy. Nutrients that are components in the body are also broken down as needed and eventually metabolized as energy. In the process of this metabolic reaction, oxygen is consumed, and carbon dioxide, water, and heat are produced. When carbohydrates, lipids, and proteins are metabolized, the ratio between the amount of oxygen consumed and the amount of carbon dioxide produced is different, which is called “RQ” [25]. Carbon dioxide production per minute (VCO_2_) and oxygen consumption per minute (VO_2_) were tested by indirect calorimetry. Total urinary excretion of nitrogen (UN) was measured as described elsewhere [6,25]. npRQ was calculated using the following formula: npRQ = (1.44VCO_2_–4.890UN) divided by (1.44VO_2_–6.04UN) [6,25].

### 2.3. SMI and ECW to TBW Ratio Using BIA

The definition of SMI was “appendicular skeletal muscle mass (kg) divided by (height (m))^2^” using BIA. Based on the current Japanese criteria, the definition of decreased SMM was: SMI < 7.0 kg/m^2^ in men and < 5.7 kg/m^2^ in women [26]. Based on the concept that excessive ECW leads to edematous state, extracellular fluid (ECF) status was defined as the ECW to TBW ratio. In healthy persons, ECW to TBW ratio can be maintained at a constant value (ECW to TBW ratio around 0.38). ECF excess was classified as follows: normal (ECW to TBW ratio < 0.390), mild overhydrated state (ECW to TBW ratio 0.390–0.399), and moderate to severe overhydrated state (ECW to TBW ratio ≥ 0.400) (Biospace Co. Ltd., Seoul, Korea) [27].

We examined the relationship between npRQ and ALBI grade and factors linked to npRQ < 0.85, which is reported to be an optimal cutoff point for the prognosis in LC patients, using univariate and multivariate analyses [6,23]. The ethics committee of our hospital (Hyogo College of Medicine Hospital) acknowledged the study (approval no. 1831). The protocol in the current study strictly observed the regulations of the Declaration of Helsinki.

### 2.4. Statistical Considerations

In continuous variables, in order to estimate between-group difference, Student’s *t* test, Mann–Whitney *U* test, Spearman’s rank coefficient *r_s_*, analysis of variance, or Kruskal–Wallis tests were employed as appropriate. Normality of data distribution to analyze the continuous variables was tested by the Shapiro–Wilk test. In categorical variables, in order to estimate between-group difference, Fisher’s exact tests or Pearson χ^2^ tests were employed as appropriate. Factors with *p* < 0.05 linked to npRQ < 0.85 in the univariate analysis were entered into the multivariate logistic regression analysis to select candidate parameters. Unless otherwise stated, data are presented as interquartile range (IQR). The threshold for statistical significance was set at *p* < 0.05. The JMP 14 (SAS Institute Inc., Cary, NC, USA) was employed to perform statistical analyses.

## 3. Results

### 3.1. Patient Baseline Characteristics

**Figure 1:**
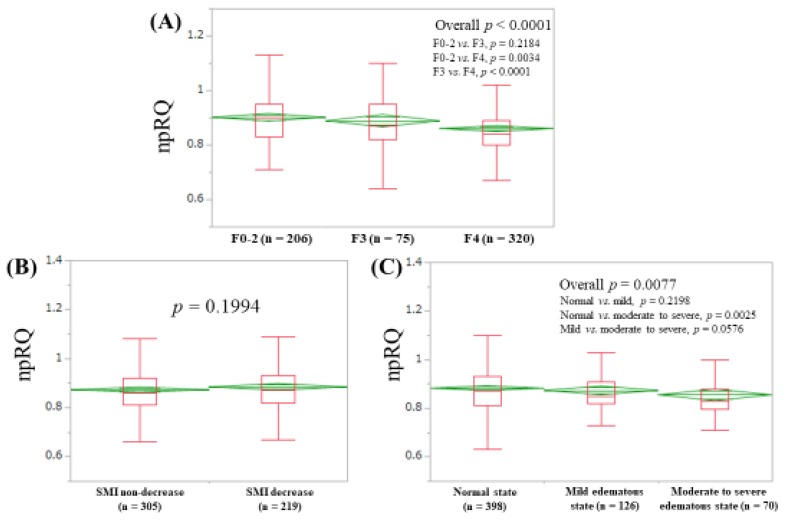
Non-protein respiratory quotient according to liver histology (**A**), skeletal muscle index (SMI) (**B**), and extracellular water (ECW) to total body water (TBW) ratio (**C**). SMI decrease indicates < 7.0 kg/m^2^ for male and < 5.7 kg/m^2^ for female. Normal state indicates ECW to TBW ratio < 0.390. Mild edematous state indicates ECW to TBW ratio 0.390–0.399. Moderate to severe edematous state indicates ECW to TBW ratio ≥ 0.400.

Baseline characteristics in our study (*n* = 601, 301 males and 300 females, median (IQR) age = 63 (54, 71) years) are presented in Table 1. The median (IQR) npRQ was 0.86 (0.81, 0.925). In total, 253 patients (42.1%) had npRQ < 0.85. The proportions of patients with npRQ < 0.85 in LC and non-LC patients were 51.9% (166/320) in LC patients and 31.0% (87/281) in non-LC patients (*p* < 0.0001). PEM, as defined by serum albumin level <3.5 g/dL and npRQ <0.85, was identified in 103 patients (17.1%: 97 patients (30.3%) in LC patients and 6 patients (2.1%) in non-LC patients). In terms of liver histology (F factor), F0 was found in 12 patients, F1 in 117, F2 in 77, F3 in 75, and F4 in 320. The median (IQR) npRQs in patients with F0-2, F3, and F4 were: 0.90 (0.83, 0.95), 0.87 (0.82, 0.95), and 0.84 (0.80, 0.89) (*p* values: F0-2 vs. F3, *p* = 0.2184; F0-2 vs. F4, *p* = 0.0034; F3 vs. F4, *p* < 0.0001 (overall *p* < 0.0001)) (Figure 1A). HCC was found in 166 patients (27.6%). There were 229 patients (38.1%) in ALBI grade 1, 328 (54.6%) in ALBI grade 2, and 44 (7.3%) in ALBI grade 3. Ascites was identified in 54 patients (9.0%). BIA testing is not appropriate for SMI in patients with massive ascites, and thus such patients were not included in this analysis. The median (IQR) npRQs in patients with SMM non-decrease (*n* = 375) and SMM decrease (*n* = 219) were 0.86 (0.81, 0.92) and 0.87 (0.82, 0.93) (*p* = 0.1194) (Figure 1B). The median (IQR) npRQs in patients with normal hydrated state (ECW to TBW ratio < 0.390, *n* = 398), mild overhydrated state (ECW to TBW ratio > 0.390 and < 0.399, *n* = 126), and moderate to severe overhydrated state (ECW to TBW ratio > 0.400, *n* = 70) were 0.87 (0.81, 0.93), 0.85 (0.82, 0.91) and 0.83 (0.7975, 0.88) (*p* values: normal vs. mild, *p* = 0.2198; normal vs. moderate to severe, *p* = 0.0025; mild vs. moderate to severe, *p* = 0.0576 (overall *p* = 0.0077)) (Figure 1C).

### 3.2. npRQ Level Among ALBI Grades 1, 2, and 3 for All Cases

**Figure 2:**
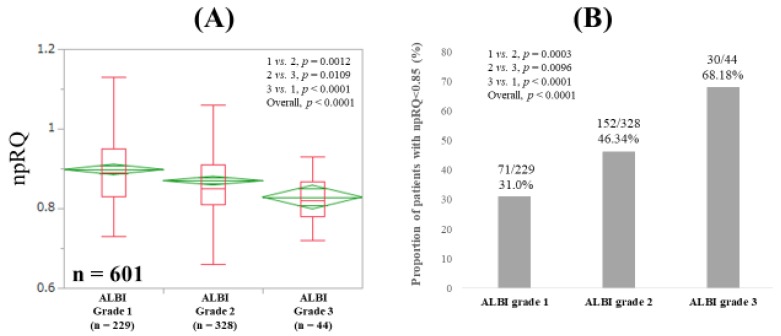
(**A**) Non-protein respiratory quotient (npRQ) value according to ALBI grade. (**B**) The proportion of npRQ < 0.85 according to ALBI grade.

The median (IQR) npRQs in ALBI grades 1, 2, and 3 for all cases were: 0.89 (0.83, 0.95), 0.85 (0.81, 0.91), and 0.82 (0.78, 0.8675) (*p* values: grade 1 vs. 2, *p* = 0.0012; grade 2 vs. 3, *p* = 0.0109; grade 3 vs. 1, *p* < 0.0001 (overall *p* < 0.0001)) (Figure 2A). While the proportions of patients with npRQ < 0.85 were: 31.0% (71/229) in ALBI grade 1, 46.34% (152/328) in ALBI grade 2, and 68.18% (30/44) in ALBI grade 3 (*p* values: grade 1 vs. 2, *p* = 0.0003; grade 2 vs. 3, *p* = 0.0096; grade 3 vs. 1, *p* < 0.0001 (overall *p* < 0.0001)) (Figure 2B).

### 3.3. npRQ Level Among ALBI Grades 1, 2, and 3 According to the LC Status

**Figure 3:**
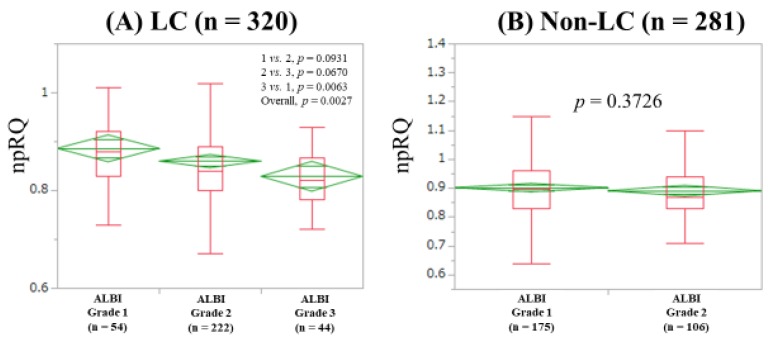
Non-protein respiratory quotient according to ALBI grade in patients with LC (**A**) and non-LC (**B**).

The median (IQR) npRQs in ALBI grades 1 (*n* = 54), 2 (*n* = 222) and 3 (*n* = 44) in LC patients were: 0.88 (0.83, 0.92), 0.84 (0.80, 0.89), and 0.82 (0.78, 0.8675) (*p* values: grade 1 vs. 2, *p* = 0.0931; grade 2 vs. 3, *p* = 0.0670; grade 3 vs. 1, *p* = 0.0063 (overall *p* = 0.0027)) (Figure 3A). The median (IQR) npRQs in ALBI grades 1 (*n* = 175) and 2 (*n* = 106) in non-LC patients were: 0.90 (0.83, 0.96) and 0.87 (0.83, 0.94) (*p* = 0.3726) (Figure 3B).

### 3.4. npRQ Level Among ALBI Grades 1, 2, and 3 According to Liver Disease Etiology

**Figure 4:**
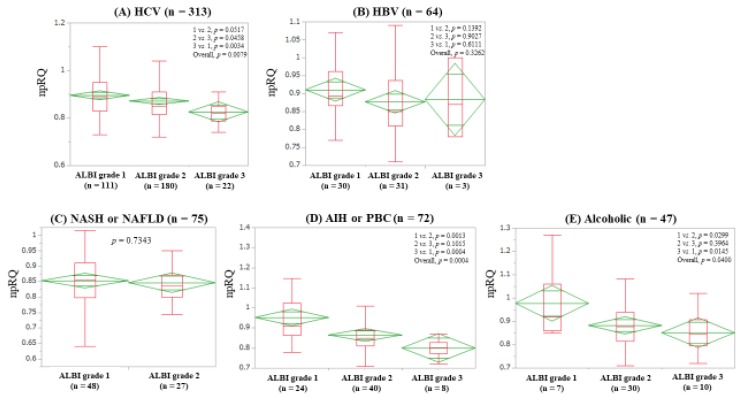
Non-protein respiratory quotient according to ALBI grade in patients with HCV (**A**) and HBV (**B**), NASH or NAFLD (**C**), AIH or PBC (**D**) and alcoholic liver disease (**E**).

The median (IQR) npRQs in ALBI grades 1 (*n* = 111), 2 (*n* = 180), and 3 (*n* = 22) in patients with hepatitis C virus (HCV) were: 0.89 (0.83, 0.95), 0.85 (0.815, 0.91), and 0.82 (0.7875, 0.85) (*p* values: grade 1 vs. 2, *p* = 0.0517; grade 2 vs. 3, *p* = 0.04458; grade 3 vs. 1, *p* = 0.0034 (overall *p* = 0.0079)) (Figure 4A). The median (IQR) npRQs in ALBI grades 1 (*n* = 30), 2 (*n* = 31), and 3 (*n* = 3) in patients with hepatitis B virus (HBV) were: 0.893 (0.8675, 0.9625), 0.85 (0.81, 0.936), and 0.87 (0.78, 1.0) (*p* values: grade 1 vs. 2, *p* = 0.1392; grade 2 vs. 3, *p* = 0.9027; grade 3 vs. 1, *p* = 0.6111 (overall *p* = 0.3262)) (Figure 4B). The median (IQR) npRQs in ALBI grades 1 (*n* = 48) and 2 (*n* = 27) in patients with non-alcoholic fatty liver disease or non-alcoholic steatohepatitis were: 0.8539 (0.80, 0.91) and 0.8354 (0.80, 0.871) (*p* = 0.7343) (Figure 4C). The median (IQR) npRQs in ALBI grades 1 (*n* = 24), 2 (*n* = 40) and 3 (*n* = 8) in autoimmune hepatitis (AIH) or primary biliary cholangitis (PBC) patients were: 0.91 (0.8625, 1.025), 0.85 (0.81, 0.89), and 0.802 (0.773, 0.828) (*p* values: grade 1 vs. 2, *p* = 0.0013; grade 2 vs. 3, *p* = 0.1015; grade 3 vs. 1, *p* = 0.0004 (overall *p* = 0.0004)) (Figure 4D). The median (IQR) npRQs in ALBI grades 1 (*n* = 7), 2 (*n* = 30), and 3 (*n* = 10) in patients with alcoholic liver disease were: 0.92 (0.86, 1.06), 0.878 (0.815, 0.941), and 0.845 (0.794, 0.9075) (*p* values: grade 1 vs. 2, *p* = 0.0299; grade 2 vs. 3, *p* = 0.3964; grade 3 vs. 1, *p* = 0.0145 (overall *p* = 0.040)) (Figure 4E).

### 3.5. npRQ Level Among ALBI Grades 1, 2, and 3 According to the Presence of HCC

**Figure 5:**
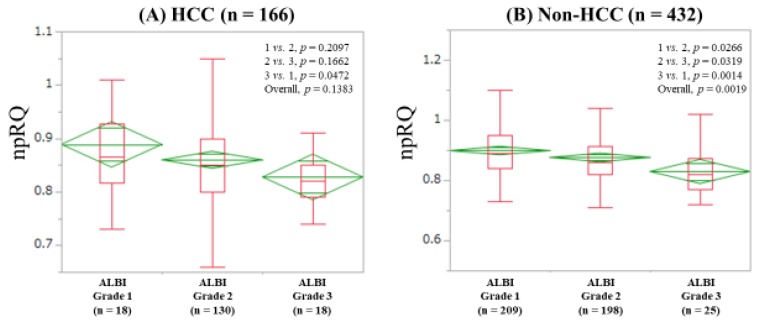
Respiratory quotient according to ALBI grade in patients with HCC (**A**) and non-HCC (**B**).

The median (IQR) npRQs in ALBI grades 1 (*n* = 18), 2 (*n* = 130), and 3 (*n* = 18) in patients with HCC were: 0.865 (0.8175, 0.9275), 0.85 (0.80, 0.90), and 0.82 (0.79, 0.85) (*p* values: grade 1 vs. 2, *p* = 0.2097; grade 2 vs. 3, *p* = 0.1662; grade 3 vs. 1, *p* = 0.0472 (overall *p* = 0.1383)) (Figure 5A). The median (IQR) npRQs in ALBI grades 1 (*n* = 209), 2 (*n* = 198), and 3 (*n* = 25) in non-HCC patients were: 0.89 (0.84, 0.95), 0.86 (0.82, 0.9125), and 0.82 (0.77, 0.8725) (*p* values: grade 1 vs. 2, *P* = 0.0266; grade 2 vs. 3, *p* = 0.0319; grade 3 vs. 1, *p* = 0.0014 (overall *p* = 0.0019)) (Figure 5B).

### 3.6. Correlation between npRQ and ALBI Score and Child-Pugh Score for All Cases

**Figure 6:**
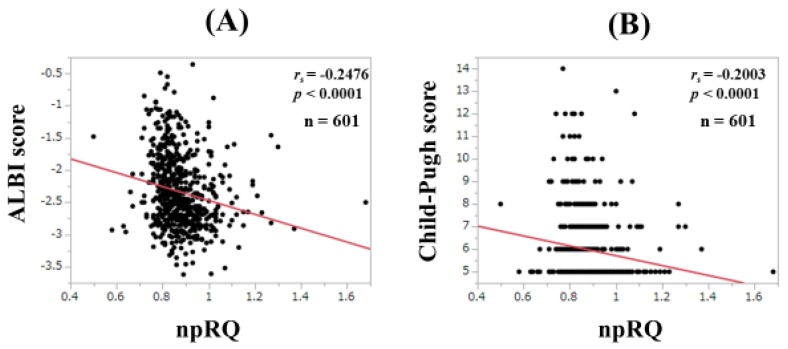
Correlation between npRQ and ALBI score (**A**) and Child-Pugh score (**B**) for all cases.

ALBI score ranged from −3.62 to −0.36 (median, −2.44). ALBI score significantly correlated with npRQ level for all cases (*r_s_* = −0.2476, *p* < 0.0001, Figure 6A) Child-Pugh score ranged from 5–14 (median, 5). Child-Pugh score significantly correlated with npRQ level for all cases (*r_s_* = −0.2003, *p* < 0.0001, Figure 6B).

### 3.7. Correlation between npRQ and ALBI Score According to Liver Disease Etiology

**Figure 7:**
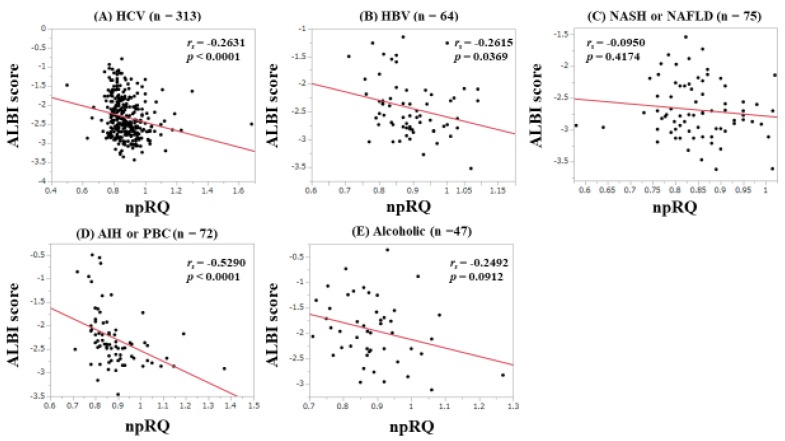
Correlation between npRQ and ALBI score according to liver disease etiology. (**A**) HCV, (**B**) HBV, (**C**) NAFLD or NASH, (**D**) AIH or PBC, and (**E**) alcoholic liver disease.

Correlation between npRQ and ALBI score according to liver disease etiology was shown in Figure 7A–E. In patients with HCV (*r_s_* = −0.2631, *p* < 0.0001), HBV (*r_s_* = −0.2615, *p* = 0.0369), and AIH or PBC (*r_s_* = −0.5290, *p* < 0.0001), significant correlation was identified.

### 3.8. Univariate and Multivariate Analyses of Factors Associated with npRQ < 0.85

Univariate analysis identified nine factors to be significantly associated with npRQ < 0.85: age (*p* = 0.0042), body mass index (*p* = 0.0259), presence of LC (*p* < 0.0001), presence of HCC (*p* = 0.0055), ALBI grade (*p* < 0.0001), prothrombin time (PT, *p* < 0.0001), platelet count (*p* = 0.0003), total cholesterol (*p* = 0.0044), and branched-chain amino acid (BCAA) to tyrosine ratio (*p* = 0.0009) (Table 2). Multivariate analysis for the nine factors (*p* < 0.05 in the univariate analyses) revealed that PT (*p* = 0.0139), ALBI grade 3 (*p* = 0.0095, and ALBI grade 1 as a reference) were significant factors linked to npRQ < 0.85 (Table 3). Corresponding hazard ratio and 95% confidence interval were listed in Table 3.

## 4. Discussion

The npRQ calculated from indirect calorimetry represents the ratio of carbohydrate to fat oxidation, and lower npRQ value is reported to be an indicator of poor prognosis in patients with LC [6]. However, as mentioned above, indirect calorimetry is time-consuming and has high cost [23,24]. Not all clinicians are familiar with the indirect calorimetry used, and it therefore does not spread to the clinical settings. Thus, identifying markers related to npRQ level in patients with CLDs seems to be of clinical importance, while the ALBI grading system has spread to clinical settings due to its convenience of use and good predictability [13,14,15,16,17,18,19,20]. We therefore conducted the current analysis. The strong point in this study was its large sample size (*n* = 601). To the best of our knowledge, this is the first study examining the relationship between npRQ and ALBI grade in CLD patients.

In our data, npRQ level was well stratified according to ALBI grade for all cases, although such tendencies were not observed in several subgroups. In addition, ALBI score significantly correlated with npRQ value, and ALBI grade was an independent predictor linked to npRQ < 0.85 along with PT in the multivariate analysis. The correlation coefficient between ALBI score and npRQ (*r_s_* = −0.2476) was stronger than that between Child-Pugh score and npRQ (*r_s_* = −0.2003). These results denoted that the ALBI grading system can be helpful for predicting npRQ to some extent. The ALBI grading system includes serum albumin [12]. Saito et al. reported that supplementation with BCAA granules improved energy metabolism and serum albumin level after radiofrequency ablation therapy in HCC patients [28]. Our results can be associated with their results. In non-LC patients, however, the ALBI grading system cannot be helpful for predicting npRQ. The fact that most non-LC patients had normal albumin and bilirubin levels may be linked to our results. In the comparison of npRQ level among ALBI grades 1, 2, and 3 in HCC patients, the overall *P* value also did not reach significance. In patients with cancer, tumor-secreted or tumor-host interactions cause imbalance of energy requirements and energy uptake [29]. In HCC patients, the presence of HCC itself may be associated with energy metabolism regardless of the severity of ALBI grade [30,31,32,33].

Hanai et al. reported that plasma FFA level was significantly correlated with npRQ value (*r* = −0.39, *p* < 0.0001) [11]. In our subgroup analyses, according to liver disease etiology, the strongest correlation between ALBI score and npRQ was found in patients with AIH or PBC (*r_s_* = −0.5290, *p* < 0.0001), which had a stronger impact on npRQ than their data [11]. In autoimmune liver diseases, ALBI grade may be helpful for predicting energy malnutrition.

As shown in Figure 1A, the difference of npRQ level in patients with F0-2 and F3 did not reach significance, whereas npRQ levels in patients with F3 and F4 were quite different (*p* < 0.0001). This appears to be an important message for clinicians. Additionally, the proportion of patients with npRQ < 0.85 in LC patients (51.9%, 166/320) was significantly higher than that in non-LC patients (31.0%, 87/281) (*p* < 0.0001). These were expected results; more importantly, more than 30% non-LC patients had npRQ < 0.85. Clinicians should be aware of these, although a decrease in npRQ level in non-LC patients was not be able to be revealed by ALBI grade, as shown in Figure 3B. PEM was identified in 97 patients (30.3%) in our LC patients. A previous report presented that PEM was observed in 27% of LC patients (*n* = 294), which was similar to our data [8].

Patients with higher SMI may exercise well, and such patients may have higher npRQ value; however, SMI did not affect npRQ in our data [9]. The clinical implication of muscle mass or muscle function on energy metabolism in patients with CLDs should be further investigated in future studies. On the other hand, npRQ level was well stratified according to the edematous state defined by ECW to TBW ratio, as demonstrated in Figure 1C. Additionally, ECW to TBW ratio had the significant negative correlation with ALBI score (*r_s_* = −0.1261, *p* = 0.0021), although it did not reach significance in the univariate analysis linked to npRQ < 0.85. Body composition analysis using BIA is particularly attractive because of its noninvasiveness [34,35,36]. ECW to TBW ratio using BIA may provide useful information for clinicians [37].

Several limitations warrant mention in this study. First, this study was a single-center cross-sectional study with a retrospective nature. Second, the number of patients with ALBI grade 3 was relatively small for analysis, although the major strength of our study was its large sample size (*n* = 601). Third, npRQ level can vary according to patient activities, creating bias. Fourth, the correlation between npRQ level and ALBI score was relatively weak, although it reached statistical significance. Caution should therefore be applied for the interpretation of our data. Nevertheless, our study results demonstrated that npRQ level in CLDs was closely associated with ALBI grade. In conclusion, ALBI grade can be a useful marker for npRQ in patients with CLDs. In CLD patients with severe ALBI grade, presence of poor npRQ should be noted.

## Figures and Tables

**Table 1:** Baseline data (*n* = 601).

Variables	Number or Median (Interquartile Range (IQR))
Age (years)	63 (54, 71)
Gender, male/female	301/300
Body mass index (kg/m^2^)	22.2 (20.2, 24.9)
Presence of LC, yes/no	320/281
Child-Pugh classification, A/B/C	447/131/23
Presence of HCC, yes/no/unknown	166/432/3
Skeletal muscle mass index (kg/m^2^), male	7.3 (6.8, 8.0)
Skeletal muscle mass index (kg/m^2^), female	5.8 (5.3, 6.3)
ECW to TBW ratio	0.385 (0.376, 0.393)
Causes of liver disease	64/313/47/75/72/30
Hepatitis B/Hepatitis C/alcohol/NAFLD or NASH/AIH or PBC/others	
Liver histology	
F0/F1/F2/F3/F4	12/117/77/75/320
ALBI grade, 1/2/3	229/328/44
npRQ	0.86 (0.81, 0.925)
Total bilirubin (mg/dL)	0.9 (0.7, 1.3)
Serum albumin (g/dL)	3.8 (3.3, 4.1)
Prothrombin time (%)	86.0 (71.9, 95.5)
Platelets (×10^4^/mm^3^)	12.4 (7.8, 19.0)
Total cholesterol (mg/dL)	160 (134.75, 188)
AST (IU/L)	40 (28.5, 62)
ALT (IU/L)	35 (23, 61.5)
HbA1c (NGSP)	5.2 (4.9, 5.7)
Branched-chain amino acid to tyrosine ratio	5.04 (3.725, 6.335)
Ascites, yes/no/unknown	54/544/3

LC, liver cirrhosis; HCC, hepatocellular carcinoma; ECW, extracellular water; TBW, total body water; NAFLD, non-alcoholic fatty liver disease; NASH, non-alcoholic steatohepatitis; AIH, autoimmune hepatitis; PBC, primary biliary cholangitis; npRQ, non-protein respiratory quotient; ALBI, albumin-bilirubin; AST, aspartate aminotransferase; ALT, alanine aminotransferase; NGSP, National Glycohemoglobin Standardization Program.

**Table 2:** Univariate analyses of factors linked to npRQ < 0.85.

Variables	npRQ ≥ 0.85 (*n* = 348)	npRQ < 0.85 (*n* = 253)	*p* Value
Age (years)	62 (51,70)	66 (56,73)	0.0042
Gender, male/female	178/170	123/130	0.5635
HBV/HCV/alcohol/NAFLD or NASH/AIH or PBC/others	43/177/32/37/43/16	21/136/15/38/29/14	0.2124
Body mass index (kg/m^2^)	21.9 (20.1, 24.4)	22.8 (20.2, 25.6)	0.0259
Presence of LC, yes/no	154/194	166/87	<0.0001
Presence of HCC, yes/no	81/266	85/166	0.0055
ALBI grade, 1/2/3	158/176/14	71/152/30	<0.0001
Prothrombin time (%)	89.6 (76.1, 98.1)	78.9 (67.6, 91.0)	<0.0001
Platelet count (×10^4^/mm^3^)	14.2 (8.7, 19.8)	9.8 (7.2, 17.2)	0.0003
AST (IU/L)	36 (27,57)	44 (30, 65)	0.0506
ALT (IU/L)	35 (23, 62.75)	35 (24, 60)	0.9233
Total cholesterol (mg/dL)	166 (136, 193.25)	153 (129.25, 180)	0.0044
HbA1c (NGSP)	5.2 (5.0, 5.6)	5.2 (4.7, 5.7)	0.1438
BTR	5.34 (4.08, 6.42)	4.605 (3.28, 6.0325)	0.0009
ECW to TBW ratio	0.384 (0.375, 0.392)	0.386 (0.377, 0.394)	0.0643
SMI (kg/m^2^), male	7.3 (6.8, 7.85)	7.35 (6.9, 8.0)	0.3515
SMI (kg/m^2^), female	5.7 (5.3, 6.3)	5.9 (5.2, 6.4)	0.1723

Data are expressed as median value (interquartile range). HBV hepatitis B virus; HCV, hepatitis C virus; NAFLD, non-alcoholic fatty liver; NASH, non-alcoholic steatohepatitis; AIH, autoimmune hepatitis; PBC, primary biliary cholangitis; AST, aspartate aminotransferase; ALT, alanine aminotransferase; NGSP, National Glycohemoglobin Standardization Program; BTR, branched-chain amino acid to tyrosine ratio; ECW, extracellular water; TBW, total body water; SMI, skeletal muscle index.

**Table 3:** Multivariate analyses of factors linked to npRQ < 0.85.

	Multivariate Analysis		
	Hazard Ratio	95% CI	*p* Value
Age	1.593	0.488–5.201	0.4407
Platelet count	0.729	0.0963–5.515	0.7593
Body mass index	0.309	0.0808–1.181	0.0861
Total cholesterol	0.636	0.185–2.185	0.4721
BTR	0.159	0.0159–1.578	0.1162
Prothrombin time	0.118	0.0215–0.647	0.0139
Presence of HCC	1.016	0.649–1.593	0.9428
Presence of LC	1.646	0.987–2.743	0.0560
ALBI			
ALBI grade 1	1.000	Reference	
ALBI grade 2	1.465	0.930–2.309	0.1000
ALBI grade 3	3.242	1.333–7.886	0.0095

CI, confidence interval; BTR, branched-chain amino acid to tyrosine ratio; HCC, hepatocellular carcinoma; LC, liver cirrhosis; ALBI, albumin-bilirubin.
